# Navigating online emotion: affective patterns and depressive traits in youth digital engagement

**DOI:** 10.3389/fpsyg.2025.1736426

**Published:** 2026-01-16

**Authors:** Chen Jin, Jinhua Yang, Zhuang Liang, Jiaqing Qiu, Rujing Zha, Zhen Yuan, Yitong Shen, Xiaochu Zhang

**Affiliations:** 1 School of Humanities, Tongji University, Shanghai, China; 2 Yancheng Party School of CPC, Yancheng, Jiangsu, China; 3 Department of Psychology, Anhui University of Chinese Medicine, Hefei, China; 4 Department of Psychology, School of Humanities and Social Science, University of Science and Technology of China, Hefei, Anhui, China; 5 Key Laboratory of Philosophy and Social Science of Anhui Province on Adolescent Mental Health and Crisis Intelligence Intervention, Hefei Normal University, Hefei, China; 6 Key Laboratory of Brain-Machine Intelligence for Information Behavior (Ministry of Education and Shanghai), School of Business and Management, Shanghai International Studies University, Shanghai, China; 7 University of Macau, Macau, China; 8 Beijing Etown Academy, Beijing, China; 9 Department of Radiology, the First Affiliated Hospital of USTC, State Key Laboratory of Eye Health, School of Life Science, Division of Life Science and Medicine, University of Science and Technology of China, Hefei, China; 10 Guizhou Key Laboratory of Artificial Intelligence and Brain-inspired Computing, College of Mathematics and Big Data, Guizhou Education University, Guiyang, China

**Keywords:** depression, digital engagement, emotional expression, social media, Weibo, youth

## Abstract

**Introduction:**

Youth digital engagement serves as a notable avenue for the expression of emotion and the construction of self among today’s youth. This study aims to examine the patterns of youth online emotional expression and their association with individual psychological traits, particularly depressive tendencies.

**Methods:**

23,966 Weibo posts published by 103 active youth users were sampled and analyzed. An integrative framework combining Russell’s Circumplex Model with multi-level thematic analysis was applied to code each post for valence, arousal, trigger type and coping strategy. Youths also completed a standard depression-screening scale; scores were used to contrast high- versus low-depressive trait sub-groups.

**Results:**

The findings reveal that youth online emotional expression overall is characterized by a self-focused nature, high pleasure, and high arousal. The study also found that individual psychological traits influence emotional expression patterns. Individuals with depressive tendencies showed a significant propensity for higher emotional arousal expression and more no-trigger expression. Furthermore, no-trigger expression plays a mediating role in their emotional expression mechanism.

**Discussion:**

The study provides an integrative framework for youth digital engagement and highlights “no-trigger” expression as a mediator in the framework. These findings can guide early detection efforts and contribute to designing targeted digital mental health supports, as well as informing guidance for families and platform managers.

## 1 Introduction

Digital platforms now weave into the fabric of everyday youth life. They are familiar spaces where young people build identities, socialize, and express themselves (Coleman and Williams, 2013; Roth et al., 2019). Youth digital engagement therefore requires sustained scholarly attention (Wood et al., 2016). Anonymity of social media provides youth with a safe emotional expression space, distinct from real life (Han and Wang, 2015). But this anonymity also obscures real distress and makes emotional problems hard to see and manage. Weibo, as an open digital platform in China, has over 500 million daily active users. Youth aged 16–24 account for more than 80% of the total user base. This makes Weibo a valuable window for observing and managing youth online behavior (Weibo Data Center, 2024).

The link between emotions and Weibo use has become a popular topic of multidisciplinary research (Bailen et al., 2019; Nesi, 2020). Existing studies employ two broad methodological approaches. First, big-data studies tap social-media APIs, text mining, and machine learning to sift through huge streams of posts and comments to identify emotion-related patterns (Table 1). Research ties depressive traits to observable signals online. For instance, a higher share of negative words and more frequent self-disclosure (Tian et al., 2018). Second, experimental studies employ questionnaires, longitudinal tracking, or experimental designs to collect individual self-report data and investigate the causal or correlational relationships between social media and depressive symptoms (Primack et al., 2021; Ye and Gao, 2025). However, these prevailing approaches are constrained by complementary limitations: Data-driven studies lack integration with standardized psychological measures or direct participant assessment, while experimental studies often overlook the nuanced relationship between the specific content, expressive styles, and structural patterns of online posts and emotion.

**Table 1:** A summary of relevant studies on Weibo emotional expression.

Literature	Research method	Sample sizes	Findings
Tian et al. (2018).	Social media data mining: 1. Keyword “depression” filtering, combined with SVM text classifier to identify depressive posts; 2. Using linguistic feature analysis (e.g., emotion word frequency)	Over 394 million posts, with 58,897 depression-related posts analyzed	Depression-related posts on Weibo exhibit distinct linguistic patterns, enabling effective identification of depressive content through computational analysis.
Wang et al. (2025)	Social media data mining: 1. Analysis of comments on Weibo account “Zoufan”; 2. Manual and BERT model annotation, classifying depression levels.	1,414,505 comments from 200,218 users, final sample of 55,339 users	Identified that high neuroticism and low extraversion are key risk factors for depression, while social support elements like family dynamics moderate its severity.
Tan et al. (2024)	Social media data mining: Sina Weibo post analysis.	30,540 posts containing	A significant correlation between unemployment rates and increased anxiety expressions on social media, with linguistic features highlighting elevated negative emotions during economic downturns.
Current Study (Jing, 2021)	Integrated mixed-methods: 1. Russell‘s Circumplex Model for dimensional coding; 2. Three-level inductive thematic analysis (coding triggers, experience, coping); 3. BDI for validation.	103 youths; 23,966 emotional Weibo posts	Revealed a three-level structural model of online emotional expression; Linked depressive tendencies to high emotional arousal and no trigger expression; Identified no trigger expression as a mediator between depressive tendencies and emotional expression patterns.

According to existing literature, a correlation exists between expressive structure and depressive tendencies. Depressive emotions are frequently associated with non-specific and decontextualized emotional processing patterns. Previous studies have found that abstract thinking impairs social problem-solving abilities, and negative association with depression (Kambara et al., 2023; Leigh et al., 2025). Additionally, Genç et al. (2023) demonstrated that reducing ruminative thinking effectively improves depression levels in adolescents. However, existing research predominantly focuses on laboratory or clinical settings, lacking an exploration of the no trigger expression patterns in youths’ natural online expressions. Adequately capturing the expression patterns require an analytical approach capable of simultaneously quantifying emotional dimensions and deciphering narrative structure.

To address these gaps, our study introduces a novel integrative framework. We combine Russell’s (1980) Circumplex Model—which provides a parsimonious, quantitative mapping of emotional valence and arousal—with a three-level inductive thematic analysis that decodes the narrative context of triggers, experience, and coping and the Beck Depression Inventory (BDI) for clinically grounded assessment. This study proposes an integrative framework that treats “no-trigger” emotional posts as a salient structural feature of youth expression on Weibo. It aims to: (1) systematically profile the content structure and characteristics of youth online emotional expression on Weibo; and (2) investigate how depressive tendencies relate to variations in these expression patterns, with a specific focus on the mediating role of no trigger expression, thereby enabling more targeted support for youth digital engagement.

## 2 Methods

### 2.1 Participants

A total of 103 active Weibo users (31 males and 72 females, aged 18–30) were recruited for the study. This study was approved by the Biomedical Ethics Committee of University of Science and Technology of China [Approval No. 2019 N(H)011]. Participants were recruited primarily through online social networks and university communities.

Interested potential participants first received a detailed online information sheet. This document outlined the study’s aims, procedures, data confidentiality measures, and their rights. Only those who subsequently completed a digital consent form advanced to the next stage.

After consenting, participants were asked to provide their Weibo usernames for data collection and to complete the BDI via a private message. The inclusion criteria required participants to have posted an average of at least 10 original Weibo posts per month over the previous year. This inclusion criterion was adopted as a necessary methodological trade-off to ensure sufficient data volume for conducting a robust, individual-level thematic analysis of emotional expression structure.

### 2.2 Data collection

After obtaining informed consent, participants provided their personal Weibo accounts for confidential research purposes only. The research then collected 5 years of publicly available posts from these accounts as the primary data source. These Weibo posts were published in a naturalistic setting without researcher intervention. This allows for a more authentic representation of the content and characteristics of youth emotional expression on Weibo. We used all post with participant authorization. The research team obscured any quoted privacy information.

The Weibo dataset comprised over 40,000 original posts from participants. We performed a two-step data cleaning process. First, posts containing only non-rendered media placeholders (e.g., for images, video, audio, red packets) were automatically filtered out. Second, posts primarily composed of structured non-textual elements—identified by conspicuous markers such as URLs (links, lottery draws, polls) and hashtags (#) for SuperTopics—were manually removed. Finally, we retained 23,966 valid emotional posts for analysis.

### 2.3 Thematic analysis

To systematically delineate the emotional expression profile of youth on social media, this study employed a thematic analysis method.

#### 2.3.1 Coding procedure

After retaining the emotion-related Weibo posts, we coded the posts line by line. Four researchers were divided into two groups (Group A and Group B). After receiving training, each group independently undertook the coding task for approximately 12,000 posts (Tian et al., 2018).

During the coding process, members within each group discussed each post individually to reach a consensus and determine the final coding category for each post.

To test the inter-coder reliability, the two groups exchanged 8.3% (2,000 posts) of their assigned total as a cross-validation sample (Group A re-coded 1,000 of Group B’s posts, and vice versa). We selected this sample from the original 23,966 posts using stratified random sampling. The Cohen’s kappa for each thematic code was as follows: (1) emotional valence—85% agreement, *κ* = 0.69; (2) emotional arousal—94% agreement, *κ* = 0.88; (3) emotional triggers—78% agreement, *κ* = 0.67; (4) emotional coping—84%, *κ* = 0.68.

#### 2.3.2 Coding framework

First, we coded all Weibo posts for basic emotions based on Russell’s Circumplex Model of Emotion. This model provides a classic dimensional framework for constructing emotional experience. It uses the pleasure dimension and the arousal dimension as its two primary axes (Russell, 1980).

**Figure 1:**
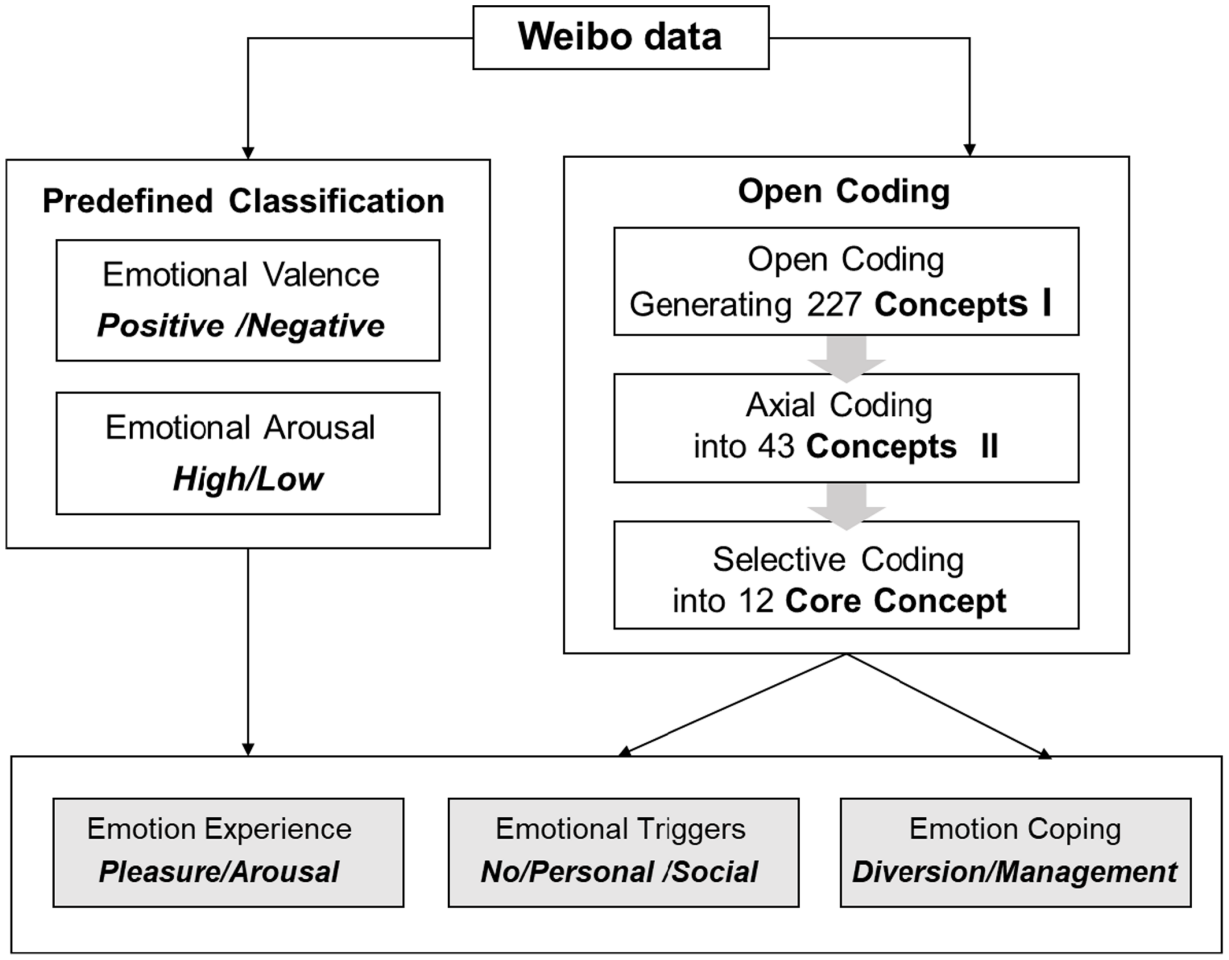
The analytical framework integrating predefined emotional dimensions and a three-level inductive coding of Weibo data.

This coding included emotional valence (positive/negative) and emotional arousal (high/low). We used a predefined classification for this coding. Simultaneously, we conducted a systematic inductive analysis of the Weibo post content itself (Figure 1, see Table 2 for coding examples). This process involved a Three-Level Inductive Coding of the textual content. Through open coding, we extracted 227 initial Concepts (Concept I). We condensed Concept I, merging duplicate and similar ones. This resulted in 43 preliminary Concepts (Concept II). Through constant comparison, we further grouped Concept II. This ultimately abstracted 12 Core Concepts reflecting the central themes of youth self-presentation. Building on the above work, we performed a theoretical integration (Figure 1, see Table 2 for coding examples). We distilled all findings into three core dimensions, which together form the final analytical framework of this study.

**Table 2:** The concept of category formed by coding.

Original post	Predefined coding	Open coding		
	Quadrant coding	Core concept	Concepts II	Concepts I
I’m so terrible, I’ll never be able to love myself.	Intense negativity	No trigger	No trigger	Self-denial
Simple joys	Mild Positive			Self-positivity
It’s the weekend!!! So happy!!!!!	Intense Positive	Personal triggers	Sharing anecdotes	Happy and Positive
“Exams, internship, CET-6, CAT-2… I’ve been so exhausted lately that my period is late… 555.”	Intense negativity		Academic and work pressure	Academic pressure
Because of one thing my supervisor said, I’ve been waiting in front of my computer until now, feeling so nervous, nervous, nervous.	Mild negativity		Interpersonal relationships	Supervisor pressure
Ugh, lost my card. So annoying.	Intense negativity		Difficulties and troubles	Annoyed by something
Huge respect to the frontline medical staff, armed police, railway workers, and airport staff. True Nightingale spirit.	Mild positive	Social triggers	Social concerns	Social event commentary
The June IELTS test has also been cancelled… Feel so sorry for the students applying this year…	Mild negativity		Phenomena around us	Sympathy for surrounding events
Cannot procrastinate any longer. Must finish the application letter this afternoon.	Mild positive	Emotional management	Problem coping	Study plan
Interpersonal relationships are always intricate and challenging. I can only blame myself for hiding too deeply while still hoping others will understand me—it’s a paradox, how is it even possible?	Mild negativity		Self-reflection	Reflection on interpersonal communication
Just thinking that there are only over ten thousand days left until retirement gives me something to look forward to.	Mild positive		Self-mockery	Retirement joke
The National College Entrance Examination is tomorrow!! I just hope I can perform normally! Or even exceed my expectations!!!!!!	Intense positive		Wish	Gaokao wish
I think I’m starting to like Yuzuru Hanyu. It turns out that one needs to have some real talent to be truly liked by others.	Mild positive	Emotional diversion	Idol/anime-manga	Adore an idol
Kafka’s writing is so captivating.	Mild positive		Hobbies	Reading sharing

Emotional intensity is coded based on the level of emotional arousal: Mild = low emotional arousal; Intense = high emotional arousal.

### 2.4 Quantitative analysis

The quantitative study aimed to investigate the relationship between emotional traits and online emotional expression. After obtaining users’ informed consent, we distributed the BDI-I via Weibo private messages. We collected 92 valid questionnaires, yielding a response rate of 89%. This study used the BDI-I to examine the impact of the emotional indicator—depressive tendencies—on emotional expression (Beck et al., 1961).

Based on their scores, we divided the participants into a depressive tendency group (*n* = 45) and a normal group (*n* = 47). We assigned individuals with a BDI-I score ≥10 to the depressive tendency group and those with a score ≤9 to the control group. The two groups showed no significant differences in gender, age, or Weibo posting volume and significant difference in BDI scores (*t* = 13.39, *p* < 0.001).

We used statistical methods such as independent samples *t*-test, correlation analysis, and mediation analysis to compare the between-group differences and explore the relationships between variables.

The mediation analysis was conducted using SPSSAU (Version 25.0) (The SPSSAU project, 2025), employing the bias-corrected bootstrap method with 5,000 resamples to estimate confidence intervals for indirect effects. Key assumptions of the analysis (e.g., linearity, absence of severe multicollinearity) were examined and met.

## 3 Results

### 3.1 Content structure and general characteristics of youth online emotional expression

**Figure 2:**
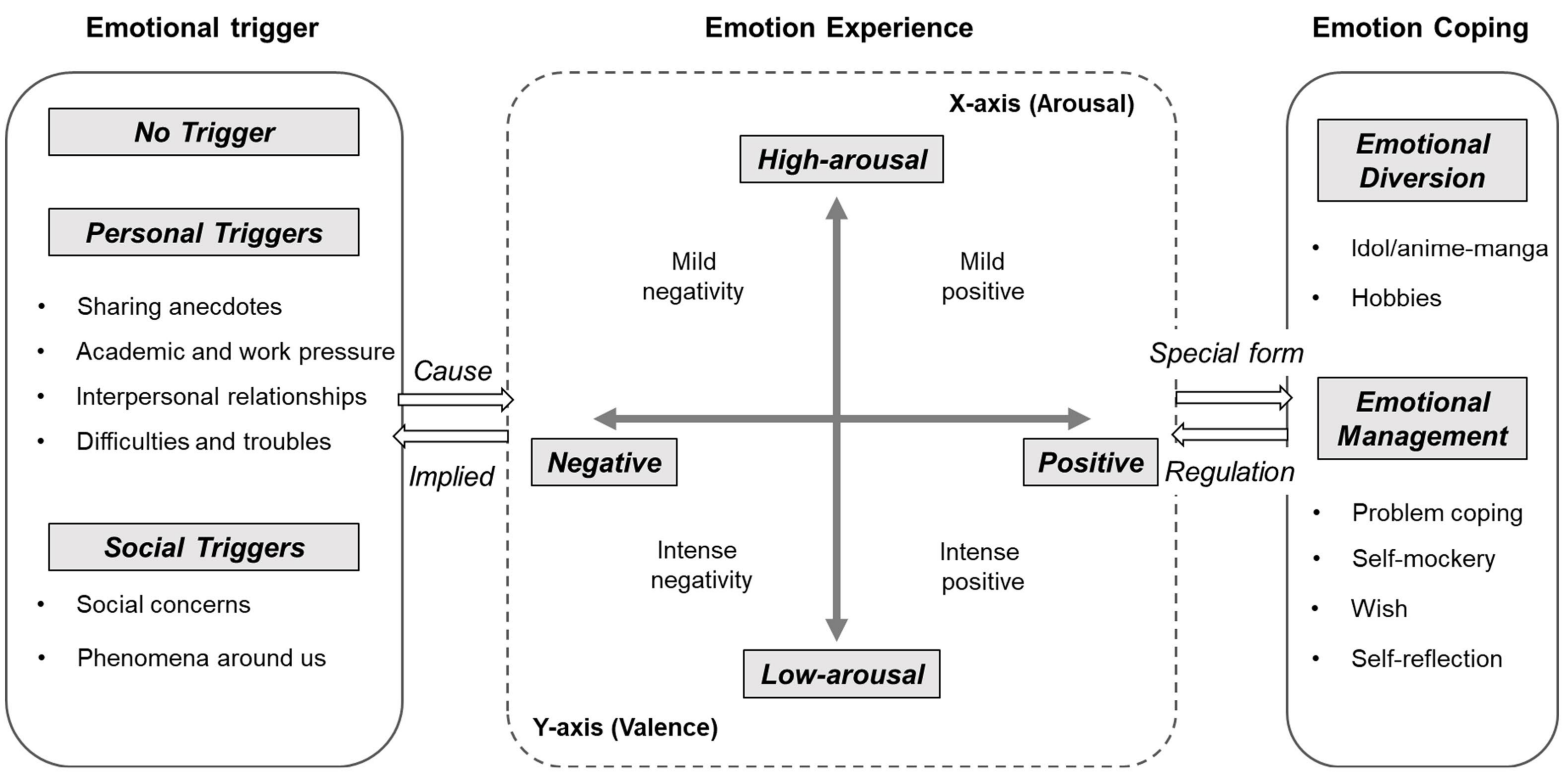
Theoretical model construction of emotional expression content of youth.

Through thematic analysis of a large-scale Weibo corpus, this study finds that youth online emotional expression forms a coherent and internally structured system. Under a three-level structure: emotion triggers, emotion experience, and emotion coping (Figure 2). It exhibits general characteristics common to the group.

#### 3.1.1 Emotion experience

*Pleasure:* According to Russell’s circumplex model of emotion, Happiness is the most common emotion. All participants used words like “happy” or “joyful” to describe positive emotional experiences. Irritation is the most common negative emotion. Eighty-four participants (81.6%) used words such as “annoyed,” “so annoyed,” “irritated,” or “sick of it” to describe their feelings. Forty-four participants (42.7%) described feelings of anger in their posts. The overall experience shows a characteristic of high pleasure (positive emotions accounted for 59%, while negative emotions accounted for 41%).

*Arousal:* Furthermore, extreme emotional expression is very common online. Strong, aggressive intentions towards oneself, others, or society are often transformed into extreme verbal statements. For example: “Every thought in my mind that cannot be obeyed fills me with aggression, I want to tear myself apart, to remove all the filthy fragments.” This demonstrates a characteristic of arousal (high arousal expressions accounted for 50%). The frequent use of exclamation marks and extreme words (e.g., “I’m so angry!”) in the posts reflects high arousal expression.

#### 3.1.2 Emotional triggers

In youth online emotional experiences, 64% described clear emotional triggers. These triggers fell into two categories: personal triggers and social triggers (Figure 2).

*No trigger* (accounting for about 33%): Youth tend to describe their emotional states directly and vividly. Participants present their own emotions through emotion-related words, phrases, emoticons, punctuation, and modal particles in their Weibo posts.

*Personal triggers* (accounting for about 64%) are the dominant type. They mainly stem from youths’ daily lives. Various frustrating daily events are the primary source of negative emotions. Physical discomfort and insomnia are typical emotional triggers. Seventy-six participants (73.8%) recorded their struggles with insomnia in their posts, for example: “Sleepless late at night, feeling awful.” The positive aspects of youths’ daily expressions primarily include sharing interesting things and achievement experiences. For instance, one participant shared: “Ate a full bowl of beef offal noodles, the spiciness was so satisfying, felt like life was reignited!”

*Social triggers* (only about 3%) refer to evaluations of social hot topics or surrounding phenomena, such as comments on public events. These findings suggest that youth emotional expression is predominantly internally oriented, focusing on personal states rather than external social commentary.

#### 3.1.3 Emotion coping

Participants’ emotional expression on social media includes not only the experience and catharsis of emotions themselves but also strategies for self-coping. This represents an extension and sublimation of emotional expression. Youth actively employ strategies to manage their emotions, primarily including:

*Emotional Diversion*: Shifting attention away from the source of negative emotions through celebrity fandom, anime and manga, or hobbies and interests. Idol and fan culture were common themes in the Weibo posts. Thematic analysis shows that posts about idol/anime-manga, and hobbies primarily express positive emotions.

*Emotional Management:* Directly addressing the emotion or problem through “self-coping” methods such as making plans, “self-mockery,” or “wish-making.” The phenomenon of wish-making is very common in the Weibo corpus. These wishes range from small lottery hopes to major life milestones. The recipients of these wishes vary widely, including teachers, idols, and anime characters.

Research indicates that emotion coping is a multidimensional construct. Coping strategies vary considerably across individuals, reflecting significant individual differences (Figure 2).

### 3.2 The effect of depressive tendencies on online emotional expression patterns

#### 3.2.1 Between-group differences in emotional expression

Building upon the established general model, we added the depressive tendencies variable for group comparison. Independent samples t-test results show that, compared to the control group, the depressive tendency group had a significantly higher proportion of both high arousal expression (*t* = −1.999, *p* = 0.049) and no trigger expression (*t* = −2.370, *p* = 0.020). This indicates that youth with depressive tendencies more habitually engage in intense, direct emotional venting on social media without anchoring their emotions to a specific event. However, the two groups showed no significant differences in dimensions such as negative emotional valence itself, emotional diversion expression, or the use of emotional management.

#### 3.2.2 Association between depressive tendencies and specific expression patterns

Building on the earlier between-group comparisons, which revealed significant differences in negative emotional expression and no trigger expression between the depressive tendency group and the control group, this study further employed grouped correlation analysis to explore the association patterns between emotional expression characteristics and depressive tendencies within each group.

After controlling for participants’ total number of Weibo posts, the analysis within the depressive tendency group yielded the following results (Table 3): BDI scores showed a significant positive correlation with emotional diversion expression (*r* = 0.354, *p* = 0.018), and a significant negative correlation with no trigger expression (*r* = −0.388, *p* = 0.009). Among correlations that did not reach the conventional threshold of statistical significance (*p* < 0.05), positive trends linking BDI scores to negative emotional expression (*r* = 0.275, *p* = 0.071) and personal triggers (*r* = 0.281, *p* = 0.065). These trends are noted but do not constitute a basis for interpretation. No significant correlations were found between BDI scores and either high arousal expression (*r* = −0.144, *p* = 0.350) or the emotional management (*r* = −0.134, *p* = 0.385).

**Table 3:** Partial correlation coefficient matrix controlling for total Weibo posts in the depressive tendency group (*n* = 45).

Variable	BDI score	No trigger	High arousal	Negative	Personal triggers	Diversion	Management
BDI Score	—						
No trigger	−0.388**	—					
High arousal	−0.144	0.623**	—				
Negative emotion	0.275†	−0.380*	−0.389**	—			
Personal triggers	0.281†	−0.821**	−0.428**	0.159	—		
Diversion	0.354*	−0.340*	−0.286†	0.379*	−0.162	—	
Management	−0.134	−0.281†	−0.298†	0.156	−0.022	−0.023	—

†*p* < 0.1; *p < 0.05; **p < 0.01.

Further analysis of the correlation patterns among the emotional expression dimensions revealed several main findings. No trigger expression showed a strong positive correlation with high arousal expression (*r* = 0.623, *p* < 0.001). Concurrently, it showed a significant negative correlation with negative emotional expression (*r* = −0.380, *p* = 0.011). Additionally, a significant negative correlation trend was observed between no trigger expression and emotional diversion expression (*r* = −0.340, *p* = 0.024).

Negative emotional expression showed a significant negative correlation with high arousal expression (*r* = −0.389, *p* = 0.009), but a significant positive correlation with emotional diversion expression (*r* = 0.379, *p* = 0.011). The emotional management showed no significant correlations with any of the other dimensions (all *p* > 0.05).

In the control group, the emotional expression patterns exhibited a distinctly different mechanism. BDI scores only showed a positive correlation with emotional management (*r* = 0.374, *p* = 0.01). No significant correlations were found with other emotional dimensions. Specifically, BDI scores were not significantly correlated with negative emotional expression (*r* = 0.102, *p* = 0.498), high arousal expression (*r* = −0.173, *p* = 0.251), emotional diversion expression (r = −0.115, *p* = 0.446), personal triggers (*r* = −0.102, *p* = 0.498), or no trigger expression (*r* = 0.065, *p* = 0.668).

#### 3.2.3 The mediating role of no trigger expression

**Figure 3:**
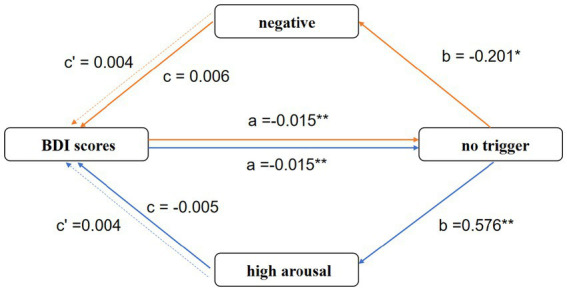
The mediating effect of no trigger expression on the relationship between BDI score and various emotional expression dimensions. Note. Path coefficients are as follows: a = effect of BDI score (X) on no trigger expression (M); b = effect of the mediator (M) on the emotional expression outcome (Y); c = total effect of X on Y; c’ = direct effect of X on Y after accounting for M. *p < 0.05, **p < 0.01.

As shown in Table 4 and Figure 3, the mediation analysis results reveal that depressive tendencies significantly influences multiple expression dimensions through the mediating role of no trigger expression.

**Table 4:** The mediating effect of no trigger expression on BDI score and multiple dimensions of emotional expression.

Model	Total effect (c)	Path a (X → M)	Path b (M → Y)	Indirect effect (a × b)	Direct effect (c’)	95% Boot CI		Relative effect (%)	Conclusion
						LL	UL		
BDI score → no trigger → negative	0.006	−0.015**	−0.201*	0.003	0.004	0.001	0.295	100.0	Full mediation
BDI score → no trigger → high arousal	−0.005	−0.015**	0.576**	−0.008	0.004	−0.457	−0.028	100.0	Full mediation

BDI, Beck Depression Inventory. Boot CI, Bias-corrected bootstrap confidence interval (5,000 resamples); LL, Lower Limit, UL, Upper Limit. The control variable “Max Rows” was included in all models. Effect Ratio Note: For models concluding ‘Full Mediation’ (i.e., a significant indirect effect with a non-significant direct effect), the proportion mediated is conventionally reported as 100%. *p < 0.05, **p < 0.01.

Specifically, the BDI score indirectly suppresses high arousal expression by significantly inhibiting no trigger expression. Second, the BDI score reduces negative emotional expression through no trigger expression. These results consistently highlight the core mediating role of no trigger expression in how depressive tendencies influences expression patterns.

In summary, the results reveal the complex transmission mechanism of emotional expression on social media in individuals with depressive tendencies, emphasizing the mediating role of no trigger expression within this process.

## 4 Discussion

### 4.1 The patterns of youth emotional expression

This study used Weibo posts from 103 young people identified in active users to analyze the content and characteristics of online emotional expression among the youth group through thematic analysis. We summarize framework of youth digital engagement and prominent characteristics of youth online emotional expression:

#### 4.1.1 Structural features of online expression

We proposed an integrated framework for analyzing digital emotion. Our approach builds upon Russell’s Circumplex Model by adding a new structure. The circumplex model offers a useful map for emotional experience (pleasure and arousal). Our three-level structure (triggers → experience→ coping) attempts to add important elements of time and personal agency. This suggests that online emotional posts are not just simple outputs of a feeling. Instead, they may include steps of regulating emotion (Gross, 2015). Therefore, young people’s social media use constitutes a cognitively engaged, proactive emotion regulation effort, rather than merely a passive reaction, and such directed expression is considered to be associated with better well-being (Burke and Kraut, 2016). We provides a process-based explanation of online emotion, identifies distinctive risks and resources within digital engagement, and links behavioral patterns to underlying psychological needs.

#### 4.1.2 Content and motivations

First, regarding emotional triggers, external issues like social problems are not the primary focus for youth. Their expression mainly aims to share self-experiences and daily life, thereby constructing their self-image. This may reflect a strategic choice to navigate external world discourse through the lens of the personal, a realm where they feel greater autonomy and control (Ryan and Deci, 2000). By sharing curated self-experiences, youth are actively engaged in identity work (Yang and Brown, 2016).

Second, youth emotional experience is characterized by high pleasure and high arousal. The highly charged posts are more likely to propagate affectively through networks, particularly among close ties (Kramer et al., 2014; Lin and Utz, 2015). Fourth, regarding emotion coping, youth construct multidimensional, divergent, and individualized coping strategies in cyberspace. Emotional diversion primarily relies on idol/anime-manga culture. These subcultures function as specialized networks of communication partners who offer shared meaning and social support (Massing-Schaffer et al., 2022; Selkie et al., 2020).

### 4.2 The mechanism and implications of depressive expression

The findings of the quantitative study indicate that the depressive tendency group showed significantly higher emotional arousal expression and no trigger expression compared to the control group. However, no significant differences were found between the two groups in negative emotional expression, emotional diversion, emotional management, or emotional triggers expression. This suggests that the emotional expression of individuals with depressive tendencies is characterized by high arousal and no trigger.

Within-group correlation analysis revealed that, in the depressive tendency group, BDI scores positively correlated with emotional diversion expression but negatively correlated with no trigger expression. This pattern unveils the association between specific emotional expression features and the severity of depression.

Viewed through Gross’ s process model (Gross, 2015), this no trigger expression pattern likely reflects a specific bottleneck in attentional deployment. This cognitive bottleneck is exemplified by the difficulty in shifting from an abstract-analytical thinking style (AAT), focused on the causes and meanings of mood, to a concrete-experiential style (CET), which is detail-oriented and contextual (Kambara et al., 2023). This pattern can be seen as a minor hiccup in early emotion regulation that gradually disconnects feelings from the events that caused them. This is consistent with the conceptualization of depressive rumination, overgeneralized, and decontextualized processing of self-relevant information (Bratman et al., 2021; Watkins and Moberly, 2009). Social media thus becomes an outlet for such abstract distress. In this sense, no trigger posts constitute observable behavioral markers that transform depressive tendencies—characterized by abstract rumination—into visible online engagement.

The correlations observed within the depressive tendency group—between BDI scores and emotional diversion expression, and between BDI scores and no trigger expression—were not found in the control group. This suggests that increasing depression severity may lead individuals to prefer immersing themselves in the world of idol/anime-manga on social media. From Erikson’s psychosocial perspective, the transition from adolescence to early adulthood is a critical period for identity formation. Within this stage, engagement with virtual worlds and identification with the personas of idols can be interpreted as a form of active identity exploration, a process in navigating the “identity versus role confusion” crisis (McLeod, 2018). This external cultural system functions as a powerfully “emotional scaffolding” providing a safe psychological space and a flexible repertoire of cultural tools and scripts. Individuals with depressive tendencies often experiencing insufficient internal regulatory resources, so they may more reliance external cultural system like idol/anime-manga (Liu et al., 2022). The previous study found idol/anime-manga are significantly associated with low self-efficacy, social anxiety, and self-harm risk (Liu et al., 2022). This indicates that monitoring an individual’s activity level within these domains could have potential value for the early identification of depression risk (Yamak and Isik, 2024).

In the control group, BDI scores only correlated positively with problem-solving strategy expression and showed no significant correlation with other emotional dimensions. This indicates that the emotional expression of individuals in the control group is more positive and constructive, as they can effectively employ emotional management to cope with emotional issues. This difference suggests that emotional expression strategies and patterns vary significantly depending on an individual’s emotional state. Individuals with depressive tendencies might alleviate negative affect by adopting more effective positive emotion regulation strategies (Gross, 2015). Social media provides a new research approach to identify potential depressive tendencies (Jing, 2021; Lachmar et al., 2017; Tian et al., 2018). This may indicate that healthy youth adopt an integrated and constructive approach; conversely, their counterparts with depressive tendencies display characteristics of escape and compensation.

No-trigger expression serves as a complete mediator through which depressive tendencies influence negative and high-arousal emotional expression on social media. Individuals with high BDI scores exhibited reduced abstract, no trigger expression. This reduction, in turn, led to a concurrent decrease in their high arousal venting behaviors. Simultaneously, the pathway showing that reduced no trigger expression leads to decreased negative emotional expression. Drawing on emotion regulation theory (Gross, 2015), the observed reduction in no-trigger expression can be understood as a form of expression suppression. Such suppression reduces outward emotional expression without diminishing the internal experience, and it also impairs memory and social function. Consequently, in the context of depression, this form of suppression may lead to the accumulation of depressive mood while simultaneously reducing negative emotional expression. Therefore, interventions should focus specifically on the no-trigger expression process, encouraging strategies like the expression of depressive emotions and cognitive reappraisal to replace maladaptive suppression.

### 4.3 Supportive strategies for digital engagement

Based on the identified architecture of youth emotional expression, and the new empirical evidence this study provides for understanding the link between depressive tendencies and specific online patterns (e.g., no trigger expression), more targeted support can be developed for youth digital engagement. The following insights are therefore drawn for family guidance and platform management.

#### 4.3.1 Implications for families: supportive and guidance strategies

For families, it helps to notice the structure of their online expression and listen for whether it includes the antecedents and consequences. Respect their “self-centered storytelling” and expressive, exaggerated styles. High arousal expression is a normal part of their emotional interaction and is not necessarily harmful. Youth subcultures should be regarded as supportive resources. The home should be a safe place to experience and express complex emotions. Parents’ primary role may be to listen and accept before offering solutions. When adolescents feel negative, parents could prompt them to describe concrete details—for example: “What happened? What did you see? What is your first step?” This Concreteness Training technique shifts attention from catastrophic meanings to actionable details, thereby reducing helplessness (Watkins and Moberly, 2009).

For families, while respecting youths’ autonomy in online expression, it is important to notice the changes in their expression patterns. When young people consistently display high arousal outbursts lacking specific context (no trigger expression) or show abnormally increased engagement with idol/anime-manga subcultures on social media, these behavioral patterns may indicate potential psychological pressure. This suggests family members should communicate with a more open attitude, focus on their emotional state. For instance, families can hold regular “subculture sharing days,” encouraging youth to introduce their favorite idols or anime content to family members. This frames such interests as a window into understanding the adolescent’s values and sources of emotional support (Leigh et al., 2025). Furthermore, families can view a noticeable increase in engagement with a subculture as an opportunity for “behavioral activation.” For instance, adolescents can be encouraged to channel their admiration for an idol into a concrete plan to learn a new skill (e.g., video editing, drawing), thereby directing emotional energy toward constructive goal pursuit (Kambara et al., 2023).

#### 4.3.2 Implications for platform design and governance

For digital platforms, interactive topics can be used to encourage structured emotional expression. Recommendation algorithms could be optimized to promote diverse and positive self-expression. It may be helpful to adopt a more structural approach to identify and evaluate content, rather than simply managing extreme or high arousal expression, and to conduct more comprehensive assessments. Platforms can design “concreteness priming” prompts through interactive topics, hashtags, or activity-based tasks. Algorithm optimization should aim to identify and appropriately amplify content that showcases problem-solving processes, specific skill learning, or constructive discussion, not just final successful outcomes. This provides users with richer, experience-based positive models (Genç et al., 2023). Platforms can establish an early warning system based on “concreteness” metrics. This system would flag high-frequency negative words and analyze the concreteness level of user expression. For accounts that persistently display high-negative, high-abstraction expression patterns, it could prioritize providing gentle support resources, such as warm-hearted content recommendations (Kirchler et al., 2025).

For digital platforms, by monitoring the distribution and changes of no trigger expression and high arousal expression, they can establish indicators for group emotional health, increase emotional attention towards individuals who post specific types of emotional content, such as idol/anime-manga themes and no trigger expressions. This may also provide important reference for early risk identification at the individual level. Platforms host a large volume of emotional expression from fan communities. They can offer more guided templates or interactive hashtags when fans post, such as #One Concrete Change My Idol Brought Me or #A Small Moment With My Idol. Platforms foster more reflective and grounded communication in these circles.

### 4.4 Limitations

#### 4.4.1 Cultural and platform specificity paired with cross-contextual testing

Our findings are derived from Chinese youth on Weibo, a context shaped by specific socio-cultural norms and platform features. To determine the generalizability of the identified emotional expression structure (e.g., the role of no trigger expression), future research could prioritize comparative studies across platforms (e.g., Twitter, TikTok) and cultures. Furthermore, we clarify that the findings based on depressive tendencies in our sample may not be directly generalization to clinical populations with diagnosed Major Depressive Disorder. Future research utilizing clinical diagnostic interviews alongside symptom scales is needed to validate and extend these findings.

#### 4.4.2 Sample bias paired with methodological diversification

The focus on active posters (≥10 posts/month) may overlook the coping strategies of less vocal users. We now discuss its potential implications—noting that the observed expression patterns (e.g., high arousal, structured coping) may be more pronounced among actively posting youth—and suggest user engagement level as an important variable for future research. Meanwhile, future work could employ stratified sampling or supplementary methods like experience sampling (ESM). This would capture a wider range of user behaviors.

#### 4.4.3 Correlational design paired with longitudinal and causal inquiry

The cross-sectional nature of our data limits our ability to draw longitudinal or causal inferences about depressive tendencies and youth online expression. Future studies could design and evaluate lightweight, theory-based platform interventions—such as prompts for emotional granularity—and track the same individuals across multiple time points to map developmental trajectories of behaviors like no trigger expression, and assess the long-term adaptive or maladaptive significance of different digital coping strategies.

## Data Availability

The datasets presented in this study can be found in online repositories. The names of the repository/repositories and accession number(s) can be found at: https://yunpan.tongji.edu.cn/link/AA0496BE97C28D4886A1B8EE78CAFFFE19.
